# Effects of Alemtuzumab on (Auto)antigen-Specific Immune Responses

**DOI:** 10.3389/fimmu.2020.563645

**Published:** 2020-10-08

**Authors:** Clara Hilger, Christine Riedhammer, Evelyn Orsó, Robert Weissert

**Affiliations:** ^1^ Department of Neurology, University Hospital of Regensburg, Regensburg, Germany; ^2^ Institute for Clinical Chemistry and Laboratory Medicine, University Hospital of Regensburg, Regensburg, Germany

**Keywords:** immunotherapy, mechanism of action, multiple sclerosis, alemtuzumab, autoantigen, T cell, CD4, MHC ligandome

## Abstract

Alemtuzumab (anti-CD52 mAb) leads to a long-lasting disease activity suppression in patients with relapsing forms of multiple sclerosis (MS). In this study, we examined the change of the immune cell repertoire and the cellular reactivity after treatment with alemtuzumab. We analyzed the number of IFN-γ–secreting cells in presence of several peptides which had been eluted from the central nervous system (CNS) of MS patients and are possible targets of autoreactive T cells in MS. The patients showed a stabilized disease activity measured in clinical parameters and lesion formation after the treatment. We detected a reduction of the number of IFN-γ–secreting cells in the presence of every tested self-antigen. The number of IFN-γ–secreting cells was also reduced in the presence of non-self-antigens. We also found a clear change in the immune cell repertoire. After an almost complete depletion of all lymphocytes, the cell specificities showed different reconstitution patterns, resulting in different cell fractions. The percentage of CD4+ T cells was clearly reduced after therapy, whereas the fractions of B and NK cells were elevated. When we evaluated the number of IFN-γ–secreting cells in relation to the number of present CD4+ T cells, we still found a significant reduction. We conclude that the reduction of IFN-γ–secreting cells by alemtuzumab is not only due to a reduction of the CD4+ T cell fraction within the peripheral blood mononuclear cell (PBMC) compartment but might also be caused by functional changes or a shift in the distribution of different subtypes in the CD4+ T cell pool.

## Introduction

Alemtuzumab has been approved for the treatment of patients with relapsing forms of multiple sclerosis (MS) since 2014. It is a humanized monoclonal antibody against the surface protein CD52, a glycosylphosphatidylinositol-anchored glycoprotein (1), which is expressed predominantly on B and T Lymphocytes, and to a lesser extent also on monocytes, macrophages and eosinophil granulocytes (2). Alemtuzumab is applied intravenously in a dose of 12 mg per day in two cycles, first for five consecutive days, one year later for three consecutive days. The drug leads to a fast depletion of CD52+ lymphocytes, whereas hematopoietic stem cells are widely undisturbed and can provide the possibility of reconstitution (3). Alemtuzumab’s effectivity has been proven in one phase II and two phase III studies where it showed superiority to treatment with interferon β-1a concerning clinical outcomes in patients with relapsing forms of MS (4–6). There are side effects of alemtuzumab that mainly relate to induction of secondary autoimmunity like autoimmune thyroid disease, immune thrombocytopenic purpura and autoimmune nephropathies (7). Also, emergence of rare autoimmune diseases has been described after treatment with alemtuzumab in patients with MS such as ANCA-associated vasculitis (8) as well as anti-GABAa receptor encephalitis (9). In this study, to receive more information about its mechanisms of action, we examined the influence of alemtuzumab on the disease activity measured in clinical parameters and magnetic resonance imaging (MRI) scans in five patients with relapsing remitting multiple sclerosis (RRMS). Importantly, we used blood samples taken of these patients to examine the change of the immune cell repertoire and of the activity of immune cells after treatment with alemtuzumab. Specifically, we assessed the influence of treatment of alemtuzumab on auto-antigen-specific Th1 T cells. Potentially, these are the disease drivers in patients with active MS (10).

## Materials and Methods

### Study Subjects

Five RRMS patients, who received alemtuzumab because of high disease activity, were enrolled in this study. Alemtuzumab was applied in two cycles, first in five consecutive days, about one year later in three consecutive days with a dose of 12 mg per day, respectively. The study duration was 36 months. At the time of the first infusion, the mean disease duration was 6 years and the mean patients age was 31 years. Subject characteristics are summarized in **Supplemental Table 1**. The study protocol was approved by the ethical committee of the University of Regensburg to the principal investigator of this study RW (approval no. 12-101-0004). All procedures performed in studies involving human participants were carried out in accordance with the ethical standards of the institutional and/or national research committee and with the 1964 Declaration of Helsinki and its later amendments or comparable ethical standards. All patients have given written informed consent.

### PBMC Isolation

Venous blood (100 ml) was drawn into sterile heparinized syringes. Peripheral blood mononuclear cells (PBMCs) were isolated by density gradient centrifugation using Lymphoprep (Stemcell Technologies, Vancouver, Canada), according to standard procedures. In two following washing steps, the cells were resuspended in RPMI medium (Thermo Fisher Scientific, Waltham, MA, USA). The PBMCs were shock-frozen in liquid nitrogen in a solution containing fetal bovine serum (Harvard Bioscience, Holliston, MA, USA) and Dimethyl Sulfoxide (DMSO, Sigma Aldrich, St. Louis, MO, USA) for later analyses. The isolation as well as the freezing and thawing of cells were performed as described (11).

### Flow Cytometry

Flow cytometric studies were performed on fresh cells as well as on frozen and thawed PBMC. Fresh cell samples were used to measure the absolute counts of T cells, B cells, and NK cells as well as the absolute counts of the subgroups of CD4+ cells and CD8+ cells within the T cell pool. For this analysis, peripheral blood samples were collected from five RRMS patients at different time points (**Supplemental Figure 1**). The absolute cell counts of patient number 1, 2, 4, and 5 at baseline were estimated based on the absolute count of leucocytes in the blood differential The patients did not receive any immunomodulatory therapy at this time, so we assumed a normal distribution of different leucocyte subsets. Because there was no specific clinical indication, the extensive measurement of lymphocyte subpopulations was not performed at this time. Subsequent analyses showed the change in the immune cell repertoire in the chronological course. Flow cytometry was also performed with the frozen and thawed PBMC to define the exact distribution of lymphocyte subpopulations in the samples for Enzyme Linked Immuno Spot Assay (ELISpot) analyses. Therefore, the frozen cells were thawed rapidly and stained for flow cytometry within two hours. Multiparametric flow cytometry of major lymphocyte subpopulations, including T, T helper, T cytotoxic, B, and NK lymphocytes, was carried out by using the BD Multitest™ 6-color TBNK platform, containing saturating amounts of fluorophore-conjugated monoclonal antibodies against CD45, CD3, CD19, CD16, CD56, CD4, and CD8 (BD Biosciences, Franklin Lakes, NJ, USA). Precise quantification of cellular subsets was performed in BD TrucountTM tubes, containing defined amounts of fluorescence microbeads (BD Biosciences). The cells were measured on a FACSCanto II flow cytometer by using the FACSCanto Clinical Software version 2.4 (BD Biosciences).

### ELISpot Assay

The ELISpot analyses were performed on isolated PBMCs which blood samples had been collected before and after the beginning of treatment (**Supplemental Figure 2**). The number of IFN-γ–secreting cells was measured in presence of various (auto)antigens. The number of IFN-γ–secreting cells was chosen as measured value, because IFN-γ is produced predominantly by Th1 cells and these seem to be of great importance in the pathogenesis of CNS autoimmunity (12). Most of the stimulation peptides we used were eluted from MHC molecules from the CNS of patients with MS (13) (**Table 1**). Two additional peptides were sequences of tetanus toxoid (14) and JC virus (15) (**Table 2**). The eluted ligands had been shown to induce elevated Th1 T cell responses in patients with active MS (10). All measurements were performed according to the manufacturer´s instructions (Mabtech, Nacka Strand, Sweden). In brief, the ELISpots plates (Mabtech), which were pre-coated with the anti–IFN-γ-monoclonal antibody mAb 1-D1K (Mabtech) were washed five times with PBS, before they were blocked with cell medium for about two hours. Then, 2*10^5 PBMCs in 100 µl of cell medium and the stimulation peptides were given to each well. For each peptide, triplicate wells were measured at a concentration of 3 µg/ml. The sample incubation was 48 h at 37°C and 5% CO_2_ in humidity. After another washing step (five times with PBS), the mAb 7-B6-1-ALP, which adheres to IFN- γ and is coupled to an alkaline phosphatase, was added. The incubation duration this time was 2 h at room temperature. After the next washing step (again five times with PBS) the spots were detected by a colorimetric reaction using 5-bromo-4chloro-3-indolyl-phosphate/nitroblue tetrazolium (Mabtech). ELISpot plates were read on an AID ELISpot Reader (Advanced Imaging Devices GmbH, Strassberg, Germany) and spots were counted using the AID ELISpot Software. Remaining artifacts counted as spots were manually removed.

**Table 1:** Overview of tested peptides that were eluted from MHC molecules from the CNS of patients with MS.

Tested eluted peptides of the CNS MHC ligandome			
Number	Name/origin of peptide	Sequence	Source HLA molecule and disease type of brain autopsy donor(s)
1	Survivin	RAIEQLAMM	DRB1*1501 (PPMS)
2	Glial fibrillary acidic protein 389-400	IRETSLDTKSVS	DRB1*1501 (SPMS)
3	Glutamate dehydrogenase	KVYNEAGVTFT	DRB1*1501 (SPMS)
4	Glutamine synthetase	LNETGDEPFQYKN	DRB1*1501 (SPMS)
			DRB1*0101 (SPMS)
			DRB1*0301 (2 patients with SPMS and one other patient with MS)
5	Neurofilament-3 (medium polypeptide)	IIEETKVEDEK	DRB1*1501 (SPMS)
			DRB1*1301 (SPMS)
6	Alpha-synuclein	YEMPSEEGYQD	DRB1*0101 (SPMS)
7	Actin	WISKQEYDESGPSIVHRK	DRB1*1501 (2 patients with SPMS)
			DRB1*0101 (SPMS and 1 other patient with MS)
			DRB1*0301 (2 patients with SPMS)
8	MBP10-27	RHGSKYLATASTMDHARH	DRB1*1501 (SPMS)
9	MBP84-94	DENPVVHFFKN	DRB1*1501 (SPMS)
			DRB1*0401 (SPMS)
10	MBP139-153	HKGFKGVDAQGTLS	DRB1*1501 (SPMS)
11	MBP95-110	IVTPRTPPPSQGKGRG	DRB1*1501 (SPMS)

HLA, human leukocyte antigen; MBP, myelin basic protein; PPMS, primary progressive MS; SPMS, secondary progressive MS.

**Table 2:** Other tested peptides.

Additional peptides			
Number	Name/origin of peptide	Sequence	Reference
12	Tetanus toxoid (tt830-844)	QYIKANSKFIGITEL	(14)
13	JC virus (VP1_34-48)	VDSITEVECFLTPEM	(15)

### Peptide Synthesis and Storage

Peptides were purchased from Pepscan (Lelystad, The Netherlands), where peptides were synthesized and purified. Purity was assessed by HPLC and mass spectrometry. Mean purity was 97.5%, ranging from 90% to 99.8%. Lyophilized peptides were solved in DMSO and stored at a concentration of 4 mg/ml at −20°C.

### Assessment of the Clinical State

The participants of the study were very well characterized in respect of their clinical course and disease management. Anamnestic questioning about their subjective well-being and neurological examinations were regularly performed before the treatment with alemtuzumab as well as after the first and second treatment.

### MRI Assessment

For the evaluation of the MRI scans of the brain, particularly the FLAIR- and T2-weighted axial images, furthermore the T1-weighted images after application of contrast medium were assessed. Also, images in sagittal and coronal direction were used for comparison. Concerning the MRI scans of the myelon, especially the sagittal scans were evaluated, but also the axial scans and proton weighted scans were considered. During the analysis, the number, localization and size of individual lesions and their development after the treatment were of special interest.

### Statistical Analysis

Statistical analyses were performed using the GraphPad Prism 7 Software (GraphPad Software, San Diego, CA, USA). The tested variables followed the Gaussian distribution only at a part of the time points (*p* differed between *p* < 0.0001 and *p* = 8714, D’Agostino Pearson omnibus normality test). Therefore, the Wilcoxon matched pairs signed rank test was used to compare the reactivities at baseline to the reactivities later. Subsequently, Bonferroni correction for multiple comparisons was used. This testing strategy was also used to compare the reactivities at the time span 350–380 days to subsequent time points. The GraphPad Prism 7 Software was also used to plot the figures.

## Results

### Alemtuzumab Decreases the Absolute Counts of Different Lymphocyte Subpopulations, Which Reorganize Differently

To receive an impression of the extent of the cell depletion immediately after the alemtuzumab infusion, blood of one patient, which was taken 7 days after the beginning of the first infusion, was examined exemplarily. Thereby, a nearly complete depletion of B and T cells was detected. The concentration of CD4+ T helper cells, CD8+ cytotoxic T cells, and B cells was not higher than 1/µl. The concentration of NK cells was about 21/µl.

**Figure 1:**
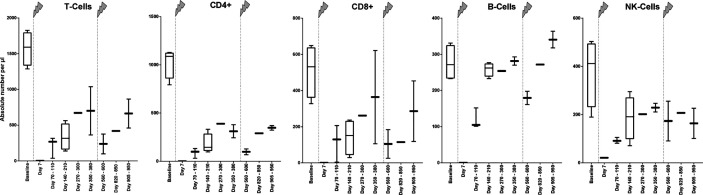
Absolute numbers of cell specificities at different time points before and during treatment with alemtuzumab of patients with MS. At baseline, absolute numbers of cell specificities of patient 1, 2, and 4 were estimated based on the counts of leucocytes in the differential blood count. The patients did not receive any therapy which influences the immune state at this time, so we assumed a normal distribution of different cell specificity fractions within the leucocytes. Because there was no clinical indication, the extensive measurement of the immune state was not performed at this time point. The arrows indicate infusion of alemtuzumab.

In the following months, several blood samples were taken to evaluate the recovery of lymphocyte subsets (**Figure 1**). During this time, every lymphocyte subpopulation began to increase again, but in different extents. The mean T cell count increased at each time point within the first year and reached a maximum of 700/µl before the second alemtuzumab infusion. The subgroup of CD4+ T cells came up to 389/µl which is less than 40% of the absolute CD4+ T cell count at baseline, whereas the subgroup of CD8+ T cells could recover about 71% of its original count with a mean absolute number of 364/µl. B cells recovered very well after their first depletion and could even exceed their baseline value within about one year, whereas NK cells did not reach their baseline value.

The next analysis, which was performed after the second infusion of alemtuzumab, showed a reduction of every cell subset. The mean absolute T cell count was 237/µl. CD4+ T cells counted 98/µl, CD8+ T cells 105/µl. Just like in the year after the first infusion, B cell counts exceeded their previous baseline numbers, whereas absolute counts of other lymphocyte subpopulations remained at lower levels during the study.

### Following Alemtuzumab Infusion, the Cellular Reactivity of PBMC on Each of the Analyzed Peptides Decreases

**Figure 2:**
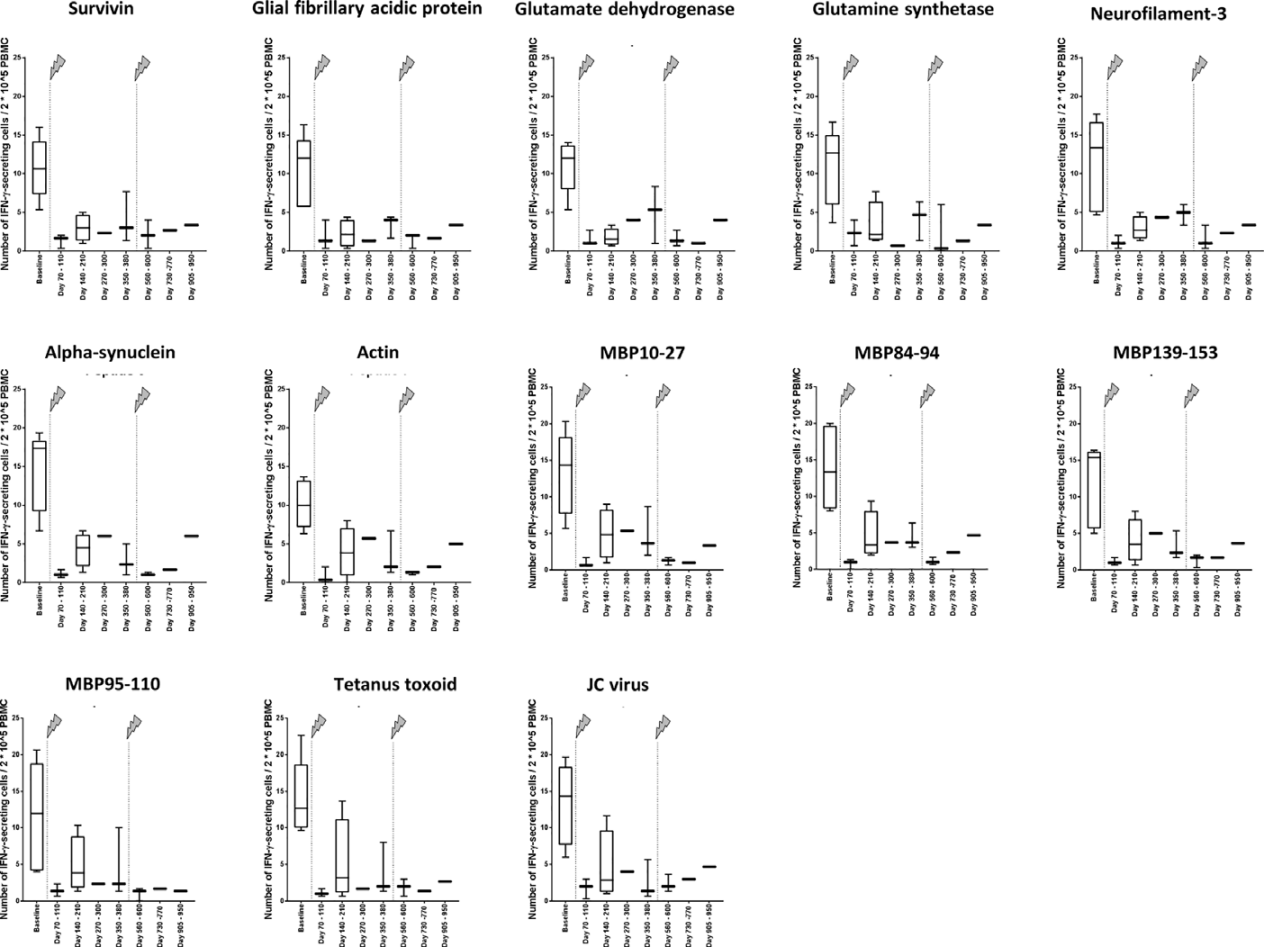
Cellular reactivity to antigens at different time points in relation to 2*10^5 PBMC. Data are shown for the absolute number of IFN-γ–secreting cells in relation to 2*10^5PBMC in the presence of different self-antigens (**Table 1**) and non-self-antigens (**Table 2**). The arrows indicate infusion of alemtuzumab.

**Figure 3:**
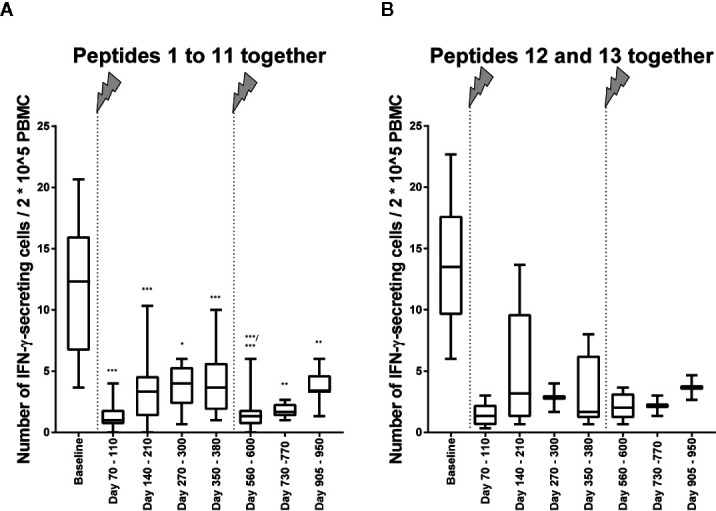
Immune reactivity to self- and non-self-antigens in relation to 2*10^5 PBMC. **(A)** Immune reactivity to peptides (self-antigens) 1 to 11 in relation to 2*10^5 PBMC (**Table 1**). Wilcoxon matched pairs signed rank test was performed to compare baseline reactivities to reactivities of other time points on the one hand and to compare reactivities of time point 5 to reactivities of subsequent time points. *P* values of this test are shown in the below. ***p < 0.001; **p < 0.01; *p < 0.05. **(B)** Immune reactivity to the non-self-antigens 12 and 13 (**Table 2**) in relation to 2*10^5 PBMC. Statistical analyses were not performed here, but the trend is illustrated. The arrows indicate infusion of alemtuzumab.

The number of IFN-γ–secreting cells was clearly lower about 3 months after the first infusion than at baseline (**Figure 2**). This result concerned all peptides 1 to 11, which have been eluted from CNS-derived MHC molecules of patients with MS as described (10, 13) and are possible targets of autoreactive cells in MS, but also the control peptides 12 and 13, JC virus (VP1_34-48) (15) and tetanus toxoid (tt830-844) (14), respectively (**Figure 2**). For all measurements, performed after the beginning of treatment, the reactivity was far less in those before the treatment. When all ligandome peptides 1 to 11 where evaluated together (**Figure 3A**), the difference between baseline and each of the other time points was significant (*p* differed between *p* < 0.001 and *p* < 0.01, Wilcoxon matched pair signed rank test and subsequent Bonferroni correction for multiple comparisons). The difference between time point 4 and time point 5 as well as the difference between time point 4 and time point 7 were significant, too (*p* < 0.01 respectively *p* < 0.05, Wilcoxon matched pair signed rank test and subsequent Bonferroni correction for multiple comparisons). Reactivities to the control peptides showed a similar trend (**Figure 3B**), but statistical analyses were not performed here because the low amounts of data.

### The Number of IFN-γ–Secreting Cells in Relation to Present CD4+ T Cells Decreases as Well

**Figure 4:**
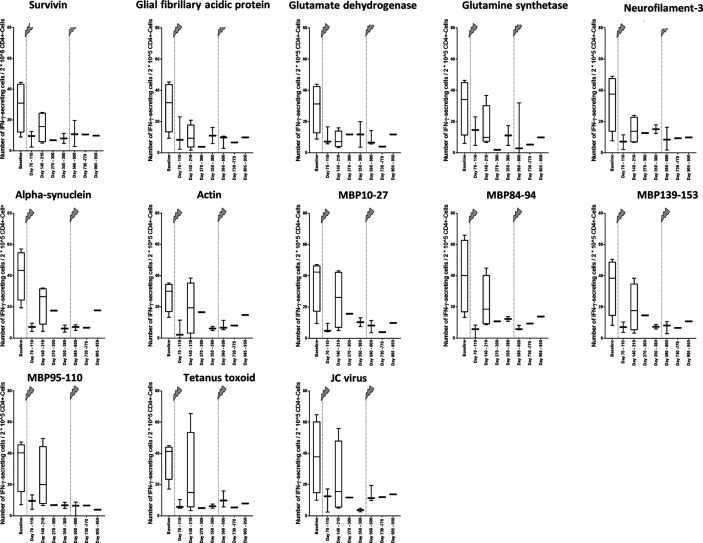
Changes of the cellular reactivity at different time points in relation to 2*10^5 CD4+ T cells. Cellular reactivity was assessed regarding different self-antigens (**Table 1**) and non-self-antigens (**Table 2**) in relation to 2*10^5 CD4+ T cells. The measured value was the number of IFN-γ–secreting cells in relation to 2*10^5 CD4+ T cells after stimulation with antigen. The arrows indicate infusion of alemtuzumab.

**Figure 5:**
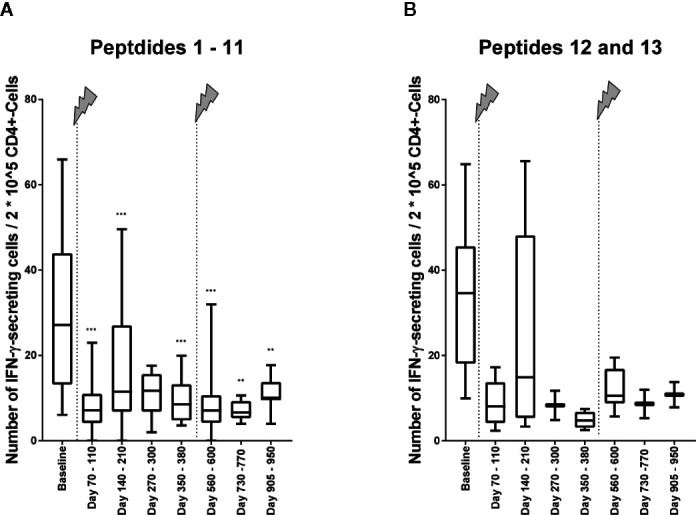
Immune reactivity to self- and non-self-antigens in relation to 2*10^5 CD4+ T cells. **(A)** Immune reactivity to peptides (self-antigens) 1 to 11 (**Table 1**) in relation to 2*10^5 CD4+ T cells. Wilcoxon matched pairs signed rank test was performed to compare baseline reactivities to reactivities of other time points on the one hand and to compare reactivities of time point 5 to reactivities of subsequent time points. P values of this test are shown in the below. ***p < 0.001; **p < 0.01 **(B)** Immune reactivity to non-self-antigens 12 and 13 (**Table 2**) in relation to 2*10^5 CD4+ T cells. Statistical analyses were not performed here, but the trend is illustrated. The arrows indicate infusion of alemtuzumab.

To find out, whether the decrease of the cellular reactivity can be explained by the change in the immune cell repertoire with lower fractions of IFN-γ–secreting CD4+ T cells or if there might also occur functional cell variations after alemtuzumab treatment, we calculated the number of IFN-γ–secreting cells in relation to 2*10^5 CD4+ T cells (**Figure 4**). In the analysis peptides 1 to 11 were evaluated together again (**Figure 5A**). We could find a decrease in the cellular reactivity, which was significant, when baseline values were compared with time points 1, 2, 4, 5, 6, or 7 (*p* values differed between *p* < 0.001 and *p* < 0.01, Wilcoxon matched-pair signed rank test and subsequent Bonferroni correction for multiple comparisons). A similar trend could be seen in presence of peptides 12 and 13 as well (**Figure 5B**), but statistical analyses were not performed.

### Alemtuzumab Treatment Stabilizes the Disease Activity

The patients enrolled in the study were characterized very well in respect of their clinical state. Their course of disease before and after the beginning of alemtuzumab treatment is described as follows: The patients showed the first signs of disease between the ages of 20 and 30. The symptoms were multiple and included, paresthesia, dysarthria, paresis and vision disorders. All five patients received alemtuzumab because of high disease activity. Before alemtuzumab treatment, four of five patients received a couple of other disease modifying therapies (**Supplemental Table 1**), which could not prevent relapses or stop disease progression. One patient had remarkably high disease activity, right from the beginning, and was therefore given alemtuzumab as a first disease modifying drug. The first infusion was given to all five patients between 10/2014 and 06/2017. In the following year, the clinical state of four patients was at least stable or even improved. One patient showed symptoms of a new relapse 3 weeks after the first infusion but stabilized after treatment with steroids. The expanded disability status scale (EDSS), which lay between 2 and 4.5 points before treatment, stayed at the same level in one case and reduced in four cases.

### Alemtuzumab Treatment Delays the Progression of CNS Lesions

In the MRI scans of the brain, which were performed before the alemtuzumab therapy started, all patients had multiple lesions, partially with contrast medium enhancement. During several months or years, the lesions enlarged, and new lesions developed. Within one year after the first infusion of alemtuzumab, the formation of new lesions stopped in four of five cases. The known lesions did not enlarge, in some cases, they even reduced in size. Furthermore, there was no evidence of contrast enhancement after application of gadolinium intravenously. Only one patient, who relapsed three weeks after the first infusion of alemtuzumab, showed new lesions after the treatment. Concerning the MRI scans of the myelon, the results were similar. After the beginning of the therapy, the known lesion did not change, and new lesions did not appear.

## Discussion

We show that the number of IFN-γ–secreting cells in presence of different self-antigens and non-self-antigens decreases after treatment of patients with MS with alemtuzumab when compared to baseline values. We chose to assess numbers of IFN-γ–secreting cells, because Th1 cells seem to be of great importance in the pathogenesis of MS (10, 16). Due to limited amounts of data sets, statistical analyses were not performed to every single peptide, but a decrease of IFN-γ–secreting cells can be seen clearly. The decrease persisted when the number of IFN-γ–secreting cells was normalized to the present CD4+ lymphocytes. Therefore, it is not only due to a decrease of the absolute numbers of CD4+ lymphocytes but could also indicate functional changes of the immune cell repertoire. The depletion of different cell types after treatment with alemtuzumab of MS patients in our study is in line with the findings in the phase II and phase III studies (4–6).

Allusions to a reduction of cellular reactivity by alemtuzumab are also provided by the analyses of T lymphocytes of patients with rheumatoid arthritis, who had been treated with alemtuzumab, because they showed a transient reduction of their proliferation rate after stimulation with anti-CD3 (17). The reaction to novel antigens and known antigens was also examined in patients with rheumatoid arthritis 12 years after treatment with alemtuzumab. Then the antibody production was in the age-specific reference range. Therefore, it was concluded that vaccinations in alemtuzumab treated patients would be reliable and guarantee protection despite lymphopenia on the long run (18).

A model with transgenic mice showed, that alemtuzumab neither impairs the cytolytic activity of natural killer cells *ex vivo* nor the capacity of macrophages for phagocytosis *in vivo* nor the antibody production of B lymphocytes to a T-independent antigen in a significant manner (19). The results of the same study also indicated a stable reactivity of T lymphocytes after alemtuzumab. In contrast to the above indicated results in patients with rheumatoid arthritis, the T lymphocytes had a normal proliferation and cytokine production after stimulation with anti-CD3 (17). Furthermore, their primary reaction to an immunization with adenovirus Ad2 was not significantly impaired (19).

The reduced number of IFN-γ–secreting cells, which was observed in the ELISpot analyses, could also be caused by a reduction of the Th1 fraction within the CD4+ lymphocyte pool, which was already seen in MS patients (20, 21). In one study, the reduction of Th1 cells was accompanied by a reduced concentration of IFN-γ in the serum (20), whereas in a mouse model, alemtuzumab did not decrease significantly the IFN-γ production per Th1 cell (19).

We did not assess the influence of depletion on T regulatory cells. An increase of Treg cells was found directly following depletion with alemtuzumab in patients with MS (22). The authors concluded that this is due to an early relative alteration of the distribution in the T cell repertoire after depletion with alemtuzumab. On the longer run there was also depletion of Treg cells in a similar manner to other types T cells (22).

In this study, we did not investigate the emergence of secondary autoimmunity in patients with MS treated with alemtuzumab (7–9). Such studies would require larger MS patient numbers and large numbers of autoantigens derived from various organs for detection of autoantigen-specific T cell responses.

Our data strongly supports the view that the effects of cellular depletion by alemtuzumab in patients with MS could be due to a strong decrease of the immune reactivity of Th1 T cells that are specific for self-antigens. Unwanted side effects like general immunosuppression are possibly partly due to a similar depletion of non-self-reactive Th1 cells.

## Data Availability Statement

The datasets generated during and analysed during the current study are available from the corresponding author on reasonable request.

## Ethics Statement

The study protocol was approved by the ethical committee of the University of Regensburg to the principal investigator of this study RW. (approval no. 12-101-0004). The patients/participants provided their written informed consent to participate in this study.

## Author Contributions

CH carried out experiments, analyzed the data, and wrote the paper. CR carried out experiments and revised the paper. EO carried out experiments and revised the paper. RW conceived the idea, planned experiments, obtained funding, supervised the project, analyzed the data, wrote, and revised the paper. All authors contributed to the article and approved the submitted version.

## Funding

This study was funded by a grant of the Regensburg Centre for Interventional Immunology (RCI) to RW. CR obtained funding by the University of Regensburg.

## Conflict of Interest

The authors declare that the research was conducted in the absence of any commercial or financial relationships that could be construed as a potential conflict of interest.

## References

[1] Xia MQ Hale G Lifely MR Ferguson MA Campbell D Packman L Structure of the CAMPATH-1 antigen, a glycosylphosphatidylinositol-anchored glycoprotein which is an exceptionally good target for complement lysis. Biochem J (1993) 293:633–40. 10.1042/bj2930633 PMC1134413 7688956

[2] Rao SP Sancho J Campos-Rivera J Boutin PM Severy PB Weeden T Human peripheral blood mononuclear cells exhibit heterogeneous CD52 expression levels and show differential sensitivity to alemtuzumab mediated cytolysis. PloS One (2012) 7:e39416. 10.1371/journal.pone.0039416 22761788 PMC3382607

[3] Malladi RK Peniket AJ Littlewood TJ Towlson KE Pearce R Yin J British Society of Blood and Marrow Transplantation. Alemtuzumab markedly reduces chronic GVHD without affecting overall survival in reduced-intensity conditioning sibling allo-SCT for adults with AML. Bone Marrow Transplant (2009) 43:709–15. 10.1038/bmt.2008.375 19029965

[4] CAMMS223 Trial Investigators Coles AJ Compston DA Selmaj KW Lake SL Moran S Alemtuzumab vs. interferon beta-1a in early multiple sclerosis. N Engl J Med (2008) 359:1786–801. 10.1056/NEJMoa0802670 18946064

[5] Cohen JA Coles AJ Arnold DL Confavreux C Fox EJ Hartung HP CARE-MS I investigators. Alemtuzumab versus interferon beta 1a as first-line treatment for patients with relapsing-remitting multiple sclerosis: a randomised controlled phase 3 trial. Lancet (2012) 380:1819–28. 10.1016/S0140-6736(12)61769-3 23122652

[6] Coles AJ Twyman CL Arnold DL Cohen JA Confavreux C Fox EJ CARE-MS II investigators. Alemtuzumab for patients with relapsing multiple sclerosis after disease-modifying therapy: a randomised controlled phase 3 trial. Lancet (2012) 380:1829–39. 10.1016/S0140-6736(12)61768-1 23122650

[7] Costelloe L Jones J Coles A Secondary autoimmune diseases following alemtuzumab therapy for multiple sclerosis. Expert Rev Neurother (2012) 12:335–41. 10.1586/ern.12.5 22364332

[8] Horisberger A Pantazou V Cuendet G Ribi C Dunet V Théaudin M ANCA-associated life-threatening systemic vasculitis after alemtuzumab treatment for multiple sclerosis. Mult Scler (2020) 1352458519895449. 10.1177/1352458519895449 32081100

[9] Maniscalco GT Mariotto S Höftberger R Capra R Servillo G Manzo V GABAa receptor autoimmunity after alemtuzumab treatment for multiple sclerosis. Neurology (2020) 2020(10):1212/WNL.0000000000010310. 10.1212/WNL.0000000000010310 32651290

[10] Riedhammer C Halbritter D Weissert R Increased immune reactivity to central nervous system-derived naturally presented peptides in patients with active multiple sclerosis. J Allergy Clin Immunol (2017) 139:694–696 e697. 10.1016/j.jaci.2016.08.015 27639936

[11] Riedhammer C Halbritter D Weissert R Peripheral Blood Mononuclear Cells: Isolation, Freezing, Thawing, and Culture. Meth Mol Biol (2016) 1304:53–61. 10.1007/7651_2014_99 25092056

[12] O’Connor RA Prendergast CT Sabatos CA Lau CW Leech MD Wraith DC Cutting edge: Th1 cells facilitate the entry of Th17 cells to the central nervous system during experimental autoimmune encephalomyelitis. J Immunol (2008) 181:3750–4. 10.4049/jimmunol.181.6.3750 PMC2619513 18768826

[13] Fissolo N Haag S de Graaf KL Drews O Stevanovic S Rammensee HG Naturally presented peptides on major histocompatibility complex I and II molecules eluted from central nervous system of multiple sclerosis patients. Mol Cell Proteomics (2009) 8:2090–101. 10.1074/mcp.M900001-MCP200 PMC2742442 19531498

[14] Panina-Bordignon P Tan A Termijtelen A Demotz S Corradin G Lanzavecchia A Universally immunogenic T cell epitopes: promiscuous binding to human MHC class II and promiscuous recognition by T cells. Eur J Immunol (1989) 19:2237–42. 10.1002/eji.1830191209 2481588

[15] Aly L Yousef S Schippling S Jelcic I Breiden P Matschke J Central role of JC virus-specific CD4+ lymphocytes in progressive multi-focal leucoencephalopathy-immune reconstitution inflammatory syndrome. Brain (2011) 134:2687–702. 10.1093/brain/awr206 21908874

[16] Riedhammer C Weissert R Antigen Presentation, Autoantigens, and Immune Regulation in Multiple Sclerosis and Other Autoimmune Diseases. Front Immunol (2015) 6:322. 10.3389/fimmu.2015.00322 26136751 PMC4470263

[17] Brett S Baxter G Cooper H Johnston JM Tite J Rapson N Repopulation of blood lymphocyte sub-populations in rheumatoid arthritis patients treated with the depleting humanized monoclonal antibody, CAMPATH-1H. Immunology (1996) 88:13–9. 10.1046/j.1365-2567.1996.d01-650.x PMC1456458 8707338

[18] Anderson AE Lorenzi AR Pratt A Wooldridge T Diboll J Hilkens CM Immunity 12 years after alemtuzumab in RA: CD5(+) B-cell depletion, thymus-dependent T-cell reconstitution and normal vaccine responses. Rheumatology (2012) 51:1397–406. 10.1093/rheumatology/kes038 22447884

[19] Turner MJ Lamorte MJ Chretien N Havari E Roberts BL Kaplan JM Immune status following alemtuzumab treatment in human CD52 transgenic mice. J Neuroimmunol (2013) 261:29–36. 10.1016/j.jneuroim.2013.04.018 23759318

[20] Zhang X Tao Y Chopra M Ahn M Marcus KL Choudhary N Differential reconstitution of T cell subsets following immunodepleting treatment with alemtuzumab (anti-CD52 monoclonal antibody) in patients with relapsing-remitting multiple sclerosis. J Immunol (2013) 191:5867–74. 10.4049/jimmunol.130192 24198283

[21] Cox AL Thompson SA Jones JL Robertson VH Hale G Waldmann H Lymphocyte homeostasis following therapeutic lymphocyte depletion in multiple sclerosis. Eur J Immunol (2005) 35:3332–42. 10.1002/eji.200535075 16231285

[22] Haas J Würthwein C Korporal-Kuhnke M Viehoever A Jarius S Ruck T Alemtuzumab in Multiple Sclerosis: Short- and Long-Term Effects of Immunodepletion on the Peripheral Treg Compartment. Front Immunol (2019) 10:1204. 10.3389/fimmu.2019.01204 31214176 PMC6558003

