# Supplementary material for: Effects of Alemtuzumab on (Auto)antigen-Specific Immune Responses
Source: Front Immunol. 2020 Oct 8;11:563645. doi: 10.3389/fimmu.2020.563645 (PMC7578345; doi:10.3389/fimmu.2020.563645)
Supplement: Supplementary file 1 [file DataSheet_1.pdf]

Patients	Age (y) at first infusion	Sex	Duration of disease at first infusion	HLA-DRB1*1501	Immunomodulatory treatment before alemtuzumab
Patient 01	30	m	2 mo	Positive	None
Patient 02	30	m	11 y	Negative	Glatiramer acetate, IFN-beta-1alpha, Natalizumab, Fingolimod, Dimethyl fumarate
Patient 03	27	m	7 y	Positive	IFN-beta-1alpha, Teriflunomide
Patient 04	35	m	6 y	Positive	Participation at a placebo-controlled study with Daclizumab, IFN-beta-1alpha, Fingolimod
Patient 05	35	f	7 y	Negative	Glatiramer acetate, IFN-beta-1-alpha, Natalizumab, Fingolimod

Supplemental Table 1. Characteristics of study participants. f, female; IFN, interferon; m, male; mo, month; y, year

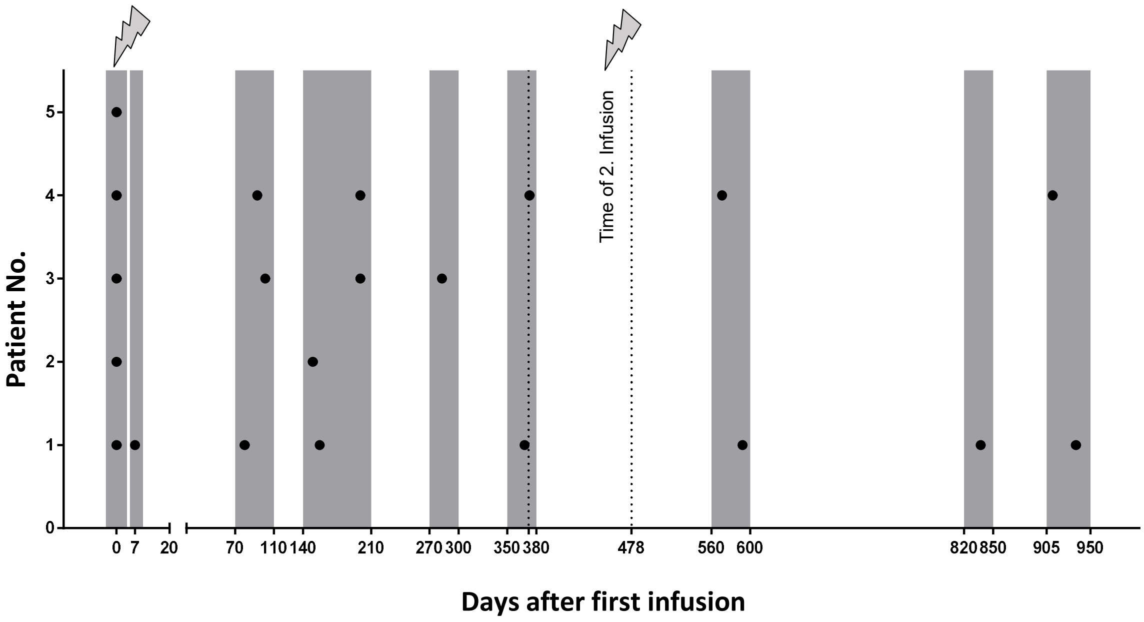

Supplemental Figure 1. Time points of flow cytometry analyses with fresh cells in relation to the first infusion of alemtuzumab. The second infusion was applied to the patients between day 372 and day 478. At the marked time points, one or more patients were taken blood samples, which were used to measure absolute numbers of cell specificities. The arrows indicate infusion of alemtuzumab.

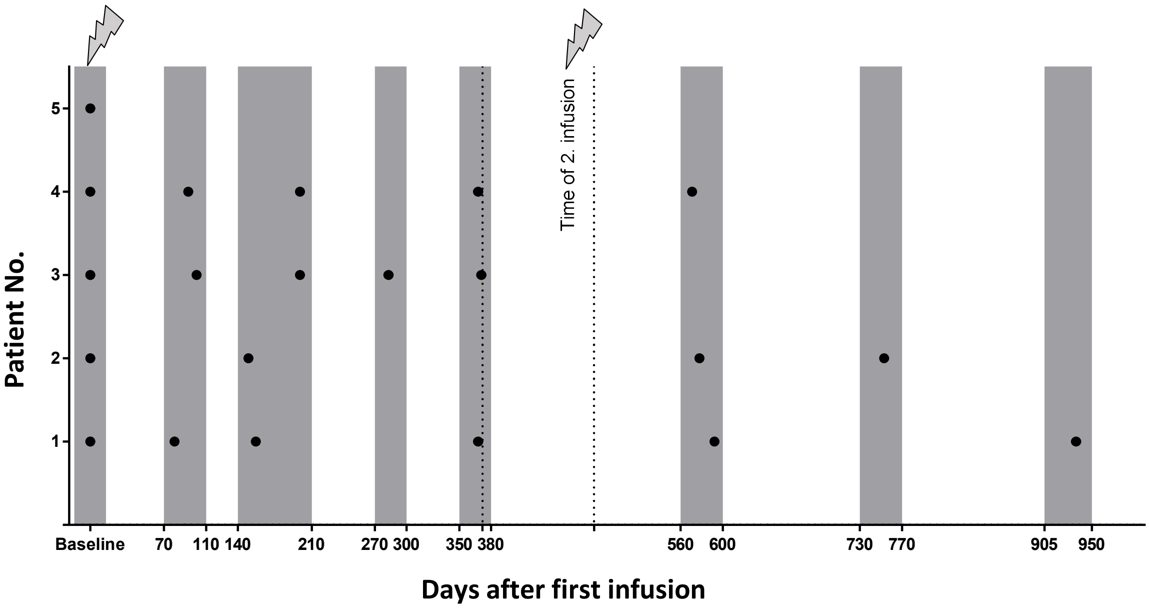

Supplemental Figure 2. Time points of the isolation and freezing of PBMCs for ELISpot analyses in relation to the first infusion of alemtuzumab. The PBMCs were thawed and then ELISpot analyses was performed. The second infusion was given to the patients between day 372 and 478. At the marked time points, one or more patients were taken blood samples, which were used to isolate PBMCs for following ELISpot analyses. The arrows indicate infusion of alemtuzumab.
